# Examining the Implementation of the Free Maternity Services Policy in Kenya: A Mixed Methods Process Evaluation

**DOI:** 10.15171/ijhpm.2017.135

**Published:** 2017-11-25

**Authors:** Eric Tama, Sassy Molyneux, Evelyn Waweru, Benjamin Tsofa, Jane Chuma, Edwine Barasa

**Affiliations:** ^1^Health Economics Research Unit, KEMRI Wellcome Trust Research Programme, Nairobi, Kenya.; ^2^Institute of Healthcare Management, Strathmore University, Nairobi, Kenya.; ^3^KEMRI Wellcome Trust Research Programme, Kilifi, Kenya.; ^4^The World Bank, Kenya Country Office, Nairobi, Kenya.; ^5^Nuffield Department of Medicine, University of Oxford, Oxford, UK.

**Keywords:** User Fee Removal, Free Maternity Care, Policy Fidelity, Policy Implementation, Kenya

## Abstract

**Background:** Kenya introduced a free maternity policy in 2013 to address the cost barrier associated with accessing maternal health services. We carried out a mixed methods process evaluation of the policy to examine the extent to which the policy had been implemented according to design, and positive experiences and challenges encountered during implementation.

**Methods:** We conducted a mixed methods study in 3 purposely selected counties in Kenya. Data were collected through in-depth interviews (IDIs) with policy-makers at the national level, health managers at the county level, and frontline staff at the health facility level (n=60), focus group discussions (FGDs) with community representatives (n=10), facility records, and document reviews. We analysed the data using a framework approach.

**Results:** Rapid implementation led to inadequate stakeholder engagement and confusion about the policy. While the policy was meant to cover antenatal visits, deliveries, and post-natal visits, in practice the policy only covered deliveries. While the policy led to a rapid increase in facility deliveries, this was not matched by an increase in health facility capacity and hence compromised quality of care. The policy led to an improvement in the level of revenues for facilities. However, in all three counties, reimbursements were not made on time. The policy did not have a system of verifying health facility reports on utilization of services.

**Conclusion:** The Kenyan Ministry of Health (MoH) should develop a formal policy on the free maternity services, and provide clear guidelines on its content and implementation arrangements, engage with and effectively communicate the policy to stakeholders, ensure timeliness of payment disbursement to healthcare facilities, and introduce a mechanism for verifying utilization reports prepared by healthcare providers. User fee removal policies such as free maternity programmes should be accompanied by supply side capacity strengthening.

## Background

Globally, maternal health outcomes have seldom improved despite improvements in other health indicators.^1^ Kenya made very limited progress towards achieving the millennium development goal (MDG) target of 147 maternal deaths per 100 000 live births. The 2014 demographic and health survey reported a national maternal mortality rate (MMR) of 362 maternal deaths per 100 000 live births,^2^ an improvement from 488 recorded in 2008, although the difference was not statistically significant.^2,3^ Ensuring pregnant women and mothers receive key maternal health interventions has been shown to be both effective and cost effective in reducing maternal mortality.^4^ However, coverage with key maternal health indicators in Kenya is still low. For example in 2014, only 58% of pregnant women attended 4 or more antenatal care (ANC) visits, only 61% of births were delivered in a health facility, and only 51% of women aged 15-49 had a postnatal check up in the first 48 hours after birth.^2^ Effective coverage with key maternal and child health interventions has been estimated to be 50.9%.^5^

The Kenyan health system is financed through a mix of prepayments and out of pocket payments. In the period 2012/2013, the Kenyan government spent about 6.1% of its total expenditure on health, equivalent to 6.8% of gross domestic product (GDP).^6^ Overall the Kenyan health system is financed by the government, private sources and donors with each contributing 33.5%, 39.8%, and 25.6% of total health expenditure respectively in 2012/2013.^6^ Households continue to be a major source of revenue with their contribution estimated at 32%.^6^

Out-of-pocket (OOP) payments associated with seeking maternal health services are a significant financial barrier to accessing maternal health services.^7^ In Kenya, OOP payments contribute about 27% of total health expenditure.^6^ 4.52% of Kenyans have been estimated to incur catastrophic health expenditures annually, with healthcare costs pushing 453 470 Kenyans into poverty every year.^8^

Many countries have adopted user fee removal policies to reduce the financial burden of accessing healthcare on households, including for specific priority services such as maternal and child healthcare.^9,10^ Kenya introduced user fees in public health facilities in 1989 as a result of poor economic performance, inadequate financial resources and international pressure.^11^ User fees for outpatient care were suspended in 1990 due to equity concerns,^12^ and re-introduced in 1992 because of budgetary constraints. Health facilities continued to charge varying user fees until 2004 when outpatient healthcare in public primary healthcare facilities (health centers and dispensaries), was declared free except for a minimal registration fee of KES 10 (US$0.1) in dispensaries and KES 20 (US$0.2) in health centers.^12,13^ Under this policy, popularly known as ‘the 10/20 policy,’ children below 5 years of age and specific priority health conditions (such as malaria, HIV/AIDS and tuberculosis) were exempt from any payments. However, an assessment of the 10/20 policy revealed that, while on paper user fees had been abolished, in practice facilities continued to charge fees.^13^ Immediately after the 2013 general election, the new government abolished any form of user fees in public dispensaries and health centres.^14^ Further, in order to increase access to facility based deliveries, the President of Kenya declared maternity services free in all public health facilities on June 1, 2013. The government committed KSh 3.8 Billion (US$3.8 million) in the 2013/2014 financial year to compensate facilities for revenue loss arising from user fees removal. This amount increased to KSh 4.2 billion (US$4.2 million) in 2014/2015 financial year and to 4.5 Million in 2015/2016 FY.^14^ Under the free maternity policy, public health facilities provide free delivery services and the government reimburses them according to utilization of services.^14^ as reported in the health information system.

User fee policies are intended to improve access and utilization of needed healthcare services, and to reduce the economic burden of disease.^15^ However evidence shows that if not properly designed and implemented, such policies might lead to mixed outcomes.^16-24^ In this paper, we present findings of a mixed methods process evaluation of the implementation of the free maternity policy in Kenya. The findings are relevant to current health systems debates locally and globally particularly regarding the design and implementation of free maternity care and/or user fee removal policies.

## Methods

### Study Setting

In 2013, Kenya transitioned into a devolved system of governance comprising of two levels: the national government and 47 semiautonomous county governments.^25^ Under devolution, the health service delivery function was transferred to county governments while the national government retained policy and regulatory functions. Across the country, health services are provided by both public (51%) and private providers who consist of for-profit and not-for-profit providers (49%).^26^ Service provision in the public sector is organized into four levels; community, primary care, county referral – managed by county governments; and national referral managed by national government.^27^ This study was conducted in the counties of Kilifi, Kajiado, and Vihiga. Table 1 outlines the characteristics of the study counties.

**Table 1:** Characteristics of Selected Counties

**Indicator**	**Kilifi**	**Kajiado**	**Vihiga**
Population (2009)	1 109 735	687 312	554 622
Share of Urban population, 2009 (%)	26%	41%	31%
Facility deliveries at 2014 (% of total deliveries)	52.6%	62.4%	50.2%
Percentage delivered by a skilled provider (2014)	52.3%	63.2%	48.6%
Percentage of women receiving ANC from a skilled provider (2014)	98.2%	96.7%	97.1%
Nurses (per 100 000 people) (2012)	37	44	40
Doctors (per 100 000 people) (2012)	5	2	4
Clinical officers (per 100 000 people) (2012)	7	7	8
Public health facilities (2012)	93	79	43
Non-governmental health facilities (2012)	6	9	4
Faith-based health facilities (2012)	13	20	10
Private health facilities (2012)	110	104	22

Abbreviation: ANC, antenatal care. Source:^2,28,29^

### Conceptual Framework

This study was conceptualized as a process evaluation.^30-32^ A process evaluation aims to understand how a policy or intervention is implemented and can provide valuable insights on why we observe the outcomes we observe.^30-32^ It provides an understanding of the elements of a policy/intervention (planned and actual at implementation), the adaptations of the policy/intervention during implementation, and the experiences and perceptions of relevant actors during implementation.^31,32^ In this study, we focused on the *emergence*, *fidelity*, and *process* of the implementation of the free maternity policy in Kenya.^30-32^ To assess *emergence*, we examined the factors that led to the decision to introduce the free maternity policy in Kenya.^30,31^ To assess *fidelity*, we examined the extent to which the policy was implemented as planned.^30,31^ A fidelity assessment compares policy design and actual implementation.^30,31^ Under policy fidelity, we therefore first examined and described the policy as originally designed and intended, and thereafter examined implementation and how implementation deviated from the design on paper. To assess *process*, we examined the perceptions of implementers about how the policy was implemented, including the perceived strengths, and weaknesses of the implementation process.^30,31^

### Study Design and Data Collection

We conducted a mixed methods study that used in-depth interviews (IDIs), focus group discussions (FGDs), facility records, and document reviews to collect data. Data collection was conducted at both the national and county levels. We selected three counties, Kajiado (in the rift Valley region of Kenya), Kilifi (in the Coastal region of Kenya), and Vihiga (in the Western region of Kenya) to achieve geographical diversity. In each county we selected 3 sub-counties taking into account rural-urban variations and in each sub-county we selected a public hospital and a public health centre for inclusion in the study. A total of 9 public hospitals and 9 public health centres were included in the study. The study was conducted 2 years after the implementation of the free maternity policy. Data collection was conducted between the months of July 2015 and January 2016.

### In-depth Interviews and Focus Group Discussions

The development of the IDI and FGD topic guides was informed by the study objective and guided by the conceptual framework. That is, we formulated questions to understand how and why the policy was introduced, the design and content of the policy, how the implementation of the policy has adhered to or differed to its design on paper, and the strengths and weakness of the policy implementation. Participants for IDIs were selected purposely based on their roles in the implementation of the policy, and by snow balling. ET and EW collected the data. Qualitative data were collected through IDIs (n = 60) at the national, county, sub-county and facility level and FGDs with community representatives in the health facility management committees of health centers (n = 10). At the national level we conducted interviews with officials from the Ministry of Health (MoH) while at the county level we interviewed managers from the county and sub-county health management team. At the facility level we interviewed the facility in-charge and staff working in the maternity departments. FGDs comprising of 5-6 participants were conducted with lay people that had been selected to represent the community in the health facility management committee (HFMC) of health centres. These community representatives comprised of a woman representative, a youth representative, a representative of the local administration, a religious leader and a representative of people with disabilities. The IDIs and FGDs explored questions related to the policy context and content, stakeholders’ perceptions of the policy, policy fidelity, implementation challenges and the impacts of implementation realities. IDIs took an average of 45-60 minutes, while the FGDs took an average of 60-90 minutes. IDIs and FGDs were recorded using a digital voice recorder, supplemented by the interviewer’s own notes. A breakdown of the study participants is provided in Table 2.

**Table 2:** Summary of Key Informants’ Interviews

**Category**	**National**	**Kilifi County**	**Kajiado County**	**Vihiga County**	**Total**
Interviews with members of the county health management teams		3	4	3	10
Interviews with members of the sub-county health management teams		5	2	3	10
Interviews with hospital managers and staff		10	8	8	26
Interviews with health center managers and staff		5	3	4	12
FGDs with community representatives		4	3	3	10
National Interviews	2				2
Total	2	27	20	21	70

Abbreviation: FGD, focus group discussion.

### Document and Record Reviews

We reviewed documents that contained information on the free maternity policy, this included official communications (circulars) between the national government and county governments, and between the county governments and health facilities. Specifically, we obtained 3 such circulars. Further, in all the 18 health facilities, we reviewed clinical records (maternity/delivery registers) to extract data on the number and type of deliveries (normal or caesarean) at the facility. We collected this data from July 2011, which was 2 years before the policy was introduced, to June 2015, which was 2 years after the policy was introduced. In each health facility, we extracted data on all monthly deliveries over the period of data collection. The facility utilization data were not intended to give statistically generalizable findings, but rather provide a descriptive view of utilization of services under the free maternity programme.

### Data Management and Analysis

Qualitative data were in the form of IDI and FGD recordings which were transcribed in English (because some responses to questions asked in English were in a mix of English and Kiswahili) and exported into NVivo 10 for management and analysis. Documents selected for review were also imported into NVivo 10 for analysis. IDI, FGD, and documents for review were analysed together. We used a framework approach to analyze qualitative data (IDI and FGD transcripts, as well as documents selected for review). Framework analysis is a process that involves identifying connections between the data collected and a pre-determined thematic framework by sifting, sorting, coding and charting collected data.^33^ This approach was adopted so as to provide findings and interpretations that are relevant to policy and also to provide pragmatic recommendations. The approach entailed 5 steps namely: familiarization, development of a thematic framework, coding, charting and finally, interpretation.^33^ To familiarize ourselves with the data, ET iteratively read through the interview transcripts, as well as documents selected for review while searching for meanings, patterns and ideas, and potential themes. EB sampled the transcripts and documents and went through the same process independently and consulted with ET and EW to agree on emerging ideas and potential themes. In the next step, ET developed a thematic framework, that took the form of a coding tree, informed by the study’s conceptual framework and the ideas and themes emerging from the first step. This process was reviewed and discussed with EB and EW. The next step involved the production of codes. Coding involves identifying, organizing and labeling chunks of data in meaningful groups.^34^ Data were coded first based on the broad themes of policy emergence, policy fidelity (policy design, ,and deviations between design and implementation), and implementation process (strengths and weakness). Under each main theme we developed sub-themes emerging from the data. Coding was conducted by ET, with support from EB and EW. Specifically, EB sampled a number of transcripts and developed codes independently. ET, EB, and EW then compared the coding and refined the final coding for the study. In the next step ET charted the coded data, a process that entailed the reorganization of coded data so as to allow the identification of emerging themes. This involved reading through coded data under each category of the thematic framework and summarizing the ideas, supported by quotes from the data.^33^ EB and EW, also checked and refined coding charts developed by ET. This process resulted in summaries of ideas on each thematic heading drawn from the data sources. In the last step (interpretation), ET critically examined the charted data under each thematic category to identify key concepts and relationships between these key concepts, as identifying messages that are relevant to policy-makers. This step was conducted in consultation with (and was reviewed by) all authors. Quantitative data were first entered into MS Excel and later exported to Stata 11 for descriptive analysis. Descriptive statistics were used to examine utilization of services.

## Results

### Emergence of the Free Maternity Policy

The emergence of the policy was motivated by technical and political needs. On the technical front, the country’s MMR had remained unacceptably high for over a decade. The high MMR was attributed to, among other factors, reduced access to key maternal health interventions such as skilled deliveries. Reduced access was seen as arising largely from financial barriers from user fees. The user fee removal policy was therefore a response to the financial barrier of access. On the political front, the policy was a fulfilment of an election pledge by the ruling government in the run-up to the 2013 national elections.

“*This [the free maternity policy] is because of the evidence which has been there that we’ve had persistently high levels of maternal mortality…. even in spite of the interventions which have been undertaken in the last 10 years...The number of mothers that are delivering in their homes has remained fairly high. Among the factors which had been cited as contributing to that was access in terms of financial access to services…and of course in order to win the hearts of Kenyans I think it was picked by the Jubilee government as a priority”* [MoH Official (13/01/2016)].

In addition to increasing financial access, the policy was also intended to address supply side factors like expansion of infrastructure, availability of qualified and skilled human resource for health and provision of essential commodities. However, due to political pressure to have the policy rolled out as soon as possible, it was decided to initially implement the user fee removal, and subsequently address supply side capacity challenges.

*“It [the free maternity policy] was actually meant to increase access to [maternal health] services at the facilities. Initially, the policy was meant to be broader, including both the removal of user fees, but also to make investments to improve public facility infrastructure, and essential commodities for provision of maternal health services. But you know sometimes you have to respond also to political needs…so what became urgent was to first remove user fee charges because that had been a commitment by the government. The other things will be addressed subsequently”* [MoH Official (13/01/2016)].

### Policy Design on Paper

A review of documents revealed that there was no formal policy document for the free maternity services policy. Rather, the national government issued several circulars to county governments, healthcare facilities and other frontline implementers (such as county, and sub-county health management teams) outlining aspects of the policy. According to these documents, maternity services were to be accessed for free in all public hospitals and health centers. Funds from general government revenues were allocated by the Ministry of Finance (MoF) to the MoH for free maternity services. The benefit package was not clear, according to respondents. The initial circular issued from the MoH, outlined that maternal deliveries including caesarean section deliveries were covered. Almost a year later, another circular was issued stating that antenatal services, delivery and post-delivery care including all complications related to delivery were to be covered. The MoH, acting as the purchaser, employed a case based mechanism to reimburse healthcare facilities a fixed amount for every delivery (whether normal or caesarean section) conducted. Specifically, county hospitals and health centers were to be reimbursed KES 5000 (US$50) and KES 2500 (US$25) respectively for every delivery conducted and reported. The two public tertiary hospitals (Kenyatta national hospital and Moi teaching and referral hospital) were to be reimbursed KES 17 000 (US$170) to account for the higher likelihood that they will handle complicated deliveries. Health facilities were required to submit reports to the MoH detailing the number of deliveries conducted in a month and the MoH would then transfer the payments due to health facilities on a quarterly basis.

As an interim arrangement, providers were to be paid through existing funds disbursement arrangements between the MoH and public healthcare facilities. These were the Health Sector Services Fund (HSSF) and the Hospital Management Services Fund (HMSF) for public primary health facilities and hospitals respectively. It was anticipated that providers will be paid through National Hospital Insurance Fund (NHIF) as more services were included into the benefit package and more providers were included into the provider network.

*“Initially it was to be deliveries… you know, we also had to respond to political need… that we design something that could be delivered as soon as possible. So, we actually proposed that for now since it’s immediate, we did have structures where the ministry was disbursing funds to the facilities. We had a system of financing hospitals which had even…there was even a committee, there was a legal notice for that; it was called the called the Hospital Management Services Fund and then the same for dispensaries and health centers under the health sector services fund. So, those are the structures we posted immediately, they will be able to do that. But this was an interim arrangement and subsequently we thought that this amount that the government was providing, would still be enough to give to... to use NHIF now to give a broader package rather than only the deliveries. So, our thinking was that, interim we use this system at the ministry, but subsequently we use NHIF”* [MoH Official (13/01/2016)].

### Deviation Between Policy on Paper and Policy in Practice (Policy (In)Fidelity)

The implementation of the free maternity policy (policy in practice) deviated from the policy design (policy on paper). While on paper antenatal service, deliveries and post-delivery care were supposed to be part of the policy’s benefit package, in practice, this was not always the case. In some facilities only deliveries were covered under the policy.

“*No, it doesn’t cover ANC or PNC (postnatal care), it is purely delivery”* [Hospital manager – County B (25/11/2015)].

**Figure 1:**
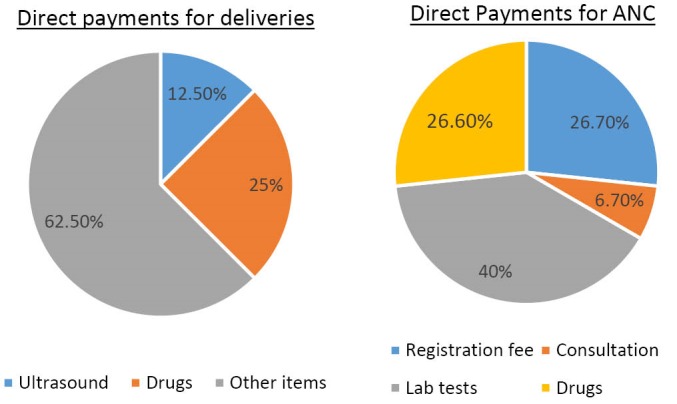
OOP Share of Services Among Clients That Incurred OOP to Access Maternity Services. Abbreviations: ANC, antenatal care; OOP, out-of-pocket.

Second, from the patient exit interviews, it emerged that the removal of user fees for maternity services was largely adhered to except for a few instances where clients were required to make direct payments to healthcare facilities. In all the counties, only 10% of the mothers who had come to deliver reported having made any OOP payment while 16% of those that had come for ANC services reported having made any form of payment. Figure 1 shows the services that clients paid for by out of pocket, and their share of total OOP costs. Supplies (cotton wool, gloves, spirit and syringes) and laboratory tests were the main drivers of OOP payments for delivery and ANC clients respectively.

### Strengths of the Implementation Processes

#### Positive Reception by Stakeholders

Overall, the policy was supported by all stakeholders across the three counties. As much as stakeholders acknowledged that there were challenges with the policy, they also felt that the policy was important and addressed a critical health problem. At the counties and health facilities, the policy was received with both excitement and apprehension. Stakeholders felt that costs were a big barrier to women’s access to facility deliveries and that this policy would increase access to maternity services and therefore reduce maternal and neonatal morbidity and mortality.

*“I believe that one [free maternity] is the best gift that the president of this country would have given to our mothers. If only some of these issues could be addressed because... I think delivery at large is very expensive. Where president giving the free maternity I think that will always decorate his legacy at the end of the day”* [County official – County C (08/09/2015)].

*“A delivery usually it would cost a patient 2500 and a caesarean section maybe 6000. So you see now and of course the deliveries are more than the caesarean section so the deliveries cater for the caesarean section. So I think it is quite adequate at our level”* [Hospital manager – County B (25/11/2015)]:

*“It was a good idea to come up with the free maternity services because yes mothers were delivering at home and there were more complications. Mothers would come in very, very late and we would lose several mothers because of complications and the fear that…there is so much fear; [that] when you go to hospital caesarean sections are high [there are so many of them]”* [Hospital manager - County A (19/08/2015)].

At the facility level, it was also felt that the reimbursement rates were adequate to compensate for the costs incurred in providing services and therefore facilities would not incur financial losses. However, there was anxiety about facilities’ preparedness to cope with the anticipated influx of patients. Stakeholders were worried about staffing, supplies, equipment and infrastructural capacity. There were also concerns about clarity of the policy especially about the exact services that were covered. As the person quoted last above went on to say:

“*It was a good move to have them come to the facility to deliver but I think we did not plan. We did not plan, we only considered mothers are coming to the facility to deliver but we did not plan for it well, yes in terms of material resources. Resources were not on the ground because we need to have made maybe we need to have a focus budget but we did not foresee. So eventually we were caught up”* [Hospital manager – County A (13/08/2015)].

#### Increased Access to Maternity Services

**Figure 2:**
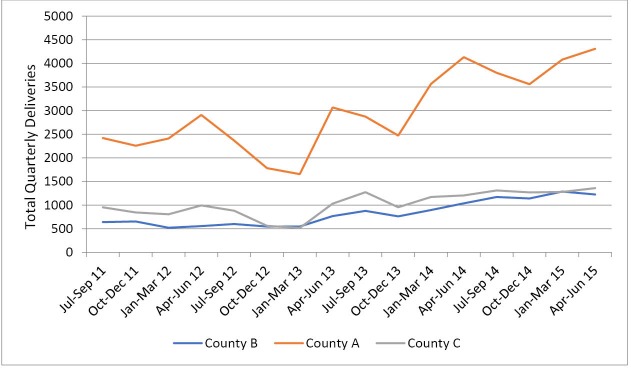
Change in Number of Facility Deliveries in the 3 Study Counties.

**Figure 3:**
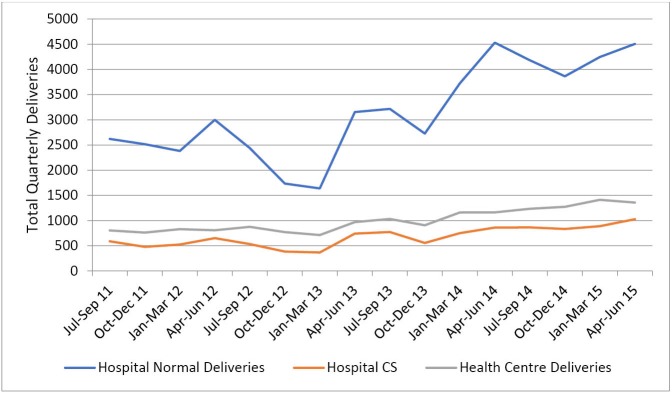
Change in Number of Facility Deliveries by Type of Delivery and Facility.

In the three counties and across all facilities, stakeholders noted that the policy had led to an increase in the number of pregnant women delivering in health facilities. In the year following the roll out of the policy, total annual reported deliveries increased by 96%, 83%, and 74% in county A, B, and C respectively. This is illustrated in Figures 2 and 3.

#### Increased Facility Revenues and Autonomy Over Resources

Under devolution, the public finance management act (PFMA) (2012) required that all revenues collected by public entities within counties be deposited in the county revenue account, rather than in the respective entities bank accounts.^35^ Hospitals, which previously had financial autonomy, and relied heavily on user fees to finance their cash budgets^36,37^ now had no control over the user fee revenues. The implementation and interpretation of the PFMA (2012) however varied across counties, especially with regard to funds that were disbursed by the MoH such as free maternity funds.

“*The reason is simple: transition. We were under the national government as a health institution all of a sudden we were pushed to devolution. Now the hospitals are not spending entities. By so I mean we don’t make direct purchases. It is the County which is making the purchases for the hospital. Us we just have a role to request. So we are not a spending entity. That is as per the devolution. Before devolution the hospital was a spending entity in the sense that it would budget on the funds that it is generating. This is revenue for the hospital and it was treated as such that it has to be used for the facility, okay? But since devolution came in its like they were coming up with their own policy on how to spend this money. So they were saying there is an Act in County Parliament which has the finance Bill which has not been passed. So actually we are receiving money which is just in the account but we can’t access it. That is a challenge”* [Hospital Manager – County A (05/08/2015)].

In County B and County C health facilities were allowed to retain free maternity funds disbursed by the national government. In these counties, facility level stakeholders acknowledged that as a result of the policy, they had more funds at their disposal. In hospitals, free maternity funds were used to pay for overheads and maintenance expenses that were critical for running the facilities. These included water, electricity and casual workers’ wages without which some hospitals would have been closed. Given that hospitals were no longer allowed to spend user fees at source, the free maternity funds emerged as a key source of finance for hospitals cash budgets.

At the health centers, free maternity funds had a big impact and in some cases were used to make major improvements to their maternity units like provision of running water, electricity and purchase of equipment and essential supplies.

*“Yeah, it has boosted us, because at least right now, the money we get from other sources can be diverted somewhere else. And our, our maternity money we can direct it to maternity. So at least it has boosted us. We have been able to procure some essential drugs, we have been able to even to, to employ some staff to assist in the services”* [Health Center manager- County B (11/11/2015)].

*“Actually you know when I looked at the figures initially I salivated because even with this rebate of 5000 per delivery we would actually collect more money than if we were charging for every delivery. Because like now before this thing came in a caesarean section of which that is maybe a much more 20% of all deliveries that one were charging maximum 10 000 even if somebody has stayed many days. And for a normal delivery it will not go beyond 2000 most of the time it would be one something, so if you take the overall you’ll find that if somebody gives you 5000 flat you’d end up collecting more money than if you charge it 2000 here and 10 000 there”* [Hospital manager – County C (24/09/2015)].

However, in County A, hospitals had completely lost financial autonomy. They therefore had no access to free maternity funds. Hospitals and health centers in this county experienced financial strain in the delivery of services since they had to provide free maternity services without compensation for revenue loss.

### Weaknesses of the Implementation Processes

#### Hurried Implementation With Inadequate Stakeholder Engagement and Communication

The political pressure to rapidly implement the policy resulted in inadequate stakeholder engagement and communication of the policy. Implementation was initiated without official guidelines and as a result the policy was understood and interpreted differently among stakeholders leading to intermittent implementation. For instance, while all stakeholders understood that deliveries are covered, there was contention about whether ANC and PNC services are covered. It was also not clear among stakeholders how often providers should be reimbursed for services offered.

*“Some circulars I had seen are saying from conception, others are talking about covering the cost during… like the last stage, in the last trimester. Others were also talking about taking care of the child all the way, but I think what we know is that it’s more active in the last trimester. From the first visit when they come, they’re not supposed to be paying any money”* [County official – County C (08/09/2015)].

*“And the ANC now… I don’t know because even we visited the governor and we tried to ask about the ANC. The mothers who are coming to us with malaria in pregnancy, they’re in the ward, but they have to pay a bill on discharge because they have not delivered. But if the same mother gave birth, it becomes free maternity now. But we were told that so long as the baby is not born, the mother has to pay the bill”* [Nurse in charge of maternity- County C (24/09/2015)].

*“Not really, sometimes we get confused: does it cover only delivery or does it run all the way from antenatal to delivery?”* [Hospital manager – County B (12/11/2015)].

*“The way I have understood the policy is that everything in maternity is free. But if you talk to the others, you will see that our understanding differs. This is because the policy has not been clearly communicated to us”* [Health Facility Management Committee FGD – County C (10/09/2015)].

*“They (National Government) should have sensitised us facility managers so that we can understand how exactly the policy was meant to work so that we can know how to implement it. They just ambushed us with the policy. It is not very clear to us, what is covered and what is excluded”* [Health Centre manager – County C (09/09/2015)].

#### Inconsistent and Unpredictable Disbursement of Funds to Facilities

In all three counties, stakeholders pointed out that reimbursements where not made on time and in most cases they were not commensurate with the deliveries conducted at the facilities. Inconsistent and unpredictable payments made it difficult for facilities to plan for the use of free maternity funds. This posed a major challenge especially for hospitals which largely relied on these funds to finance services that are essential to running of the facility.

*“The process has been very intermittent sometimes it doesn’t come in time. Sometimes we don’t even know when it will come. So, it is not something you can’t actually... And that is the biggest weakness of that program. Sometimes they do not even reimburse the whole amount. So like the facilities cannot determine how much they are going to get at the end of the quarter. Because the money was supposed to be quarterly, reimbursements. But sometimes it goes even up to six months before they reimburse. And that has put a strain in the facilities. There are facilities that are still being owed a lot of money, and we don’t know when it’s going to be paid. So more of communication, a bit of transparency and predictability, I think should actually come in, in free maternity”* [County official – County C (08/09/2015)].

*“The money comes erratically and it does not match the demand. The demand has increased but when the money comes it comes after a long time and it finds when the facility is dry so it does not really help in planning”* [Health Facility Management Committee FGD – County B (16/11/2015)].

*“One thing I would urge is at least let the free maternity funds flow as they should. If they say it is quarterly let it be quarterly, if they say it is after every two months let it be after two months and let them stick to the Ksh 2500 they said. If for example I take the 94 deliveries for last month multiply by 2500, I expect good money but they will not send the whole amount”* [Health Centre manager – County C (17/09/2015)].

Further, stakeholders raised concerns about the basis of reimbursement by MoH to health facilities. The fact that reimbursements were based on number of deliveries conducted while the benefit package included services other than just deliveries was highlighted. Stakeholders felt that this system was flawed because health facilities were paid only if a pregnant woman delivered there. They expressed concern that the health facility could lose revenues if a pregnant woman or mother accessed ANC services or PNC services in a facility where she did not deliver.

*“We have seen a challenge like even in my district hospital here, whereby the mothers are not coming to us. At times they don’t come to us. They go to Hospital X in town but the doctor there asks for ultrasound. And the mother is told, instead of paying you go to the district hospital, you get the ultra sound and then you come back to us. So you see now you become like you are offering services to the private clinic”* [County official – County B (9/12/2015)].

#### Inadequate Facility Capacity to Match Increased Utilization

While access to services had been improved and there were more pregnant women delivering in health facilities, no steps were taken to increase the health facilities’ capacity to enable them adequately cope with the increased numbers of clients. First, facility staff now had to handle more clients, which led to increased workloads leading to reported burnouts. Increased workloads impacted negatively on staff motivation and in some instances, nurses were hesitant about working in the maternity departments. Secondly, increased utilization strained facilities’ physical capacity. For instance, due to limited ward space, hospitals had been forced to fit many beds in small spaces leading to congestion and in some cases mothers shared beds. Consequently, mothers were discharged earlier than usual in order to ease congestion in the maternity wards. Moreover, the increased utilization in an environment of limited capacity compromised quality of care and some staff admitted that they were not able to give mothers the same attention they used to before the policy was introduced.

*“Yes, there are challenges like initially when it started and the number started going up, the human resource was not looked into… when people heard they came in numbers but the staff who were there they were of course strained, a lot of stress, burnouts and you can imagine what can come up from a burnout [suggesting poor quality of care]. So even the maternity staff it was not easy to contain them, yeah. It had reached a point they wanted to strike as a department. Nobody was willing to work there because if you compare to other departments there is so much work and you are paid the same. It’s straining because it involves a lot of bending and lifting, lifting mothers, so some were complaining of backaches…[that] they cannot stand”* [Hospital Nurse – County A (13/08/2015)]:

*“Now if you come or you happen to come on a Monday, you will find there are maybe 3 patients per bed. That means we have over utilized the service. So the general effect has been overutilization of the health facility or rather [it is] overstretched. Actually the facility is overstretched. There is no…it doesn’t have the capacity to handle its clients. Three patients sleeping on 1 bed with their babies, those are…that means 6 people on 1 bed. That is the biggest challenge. Sometimes in the, in the hospital the wards are full so we have to like discharge mothers earlier to create some space”* [Hospital manager – County A (05/08/2015)].

*“We do not have enough ward space. We would like a new maternity ward constructed to accommodate the increased number of mothers coming to deliver in this facility”* [Health Facility Management Committee FGD – County A (17/08/2015)].

*“Workload has increased a lot, like you currently as you can see it is just me and the sister who are in the facility. If there were no students to help us I don’t know how we would manage. There is too much workload meaning we get burnouts because of the understaffing I told you”* [Health Centre manager – County A (06/08/2015)].

#### Weak Governance and Accountability Structures

Health facilities were supposed to provide service utilization reports, which were used to determine the amount that they would be compensated. However, there was no system of verifying whether the deliveries reported were accurate. This increased the chances of health facilities gaming the system by over reporting utilization. Lack of policy guidelines that explicitly defined the roles of various stakeholders and how they were to be held accountable in implementing the policy was also cited as a weakness of the policy.

*“It is a weakness of the policy because everything was supposed to be put now on paper properly so that all the issues could be cleared in that... policy, which was never done”* [MoH official (13/01/2016)].

*“The challenges and this one I may not tell specific points… but the national government is also concerned that some counties or some facilities are cooking up figures. What they send to Nairobi and what is in the DHIS [District Health Information System]. There’s a lot of disparity, and that is a big indictment to our self. To me that I believe it is a challenge that both the county and the national government need to put a clear system. Such that when you report 20 deliveries, if somebody goes into DHIS, will find they’re actually 20 and not 10”* [County Official – County C (07/09/2015)].

## Discussion

The free maternity policy in Kenya is considered one of the key reforms aimed at accelerating the country’s progress towards universal health coverage (UHC). The Kenyan experience brings to the fore a number of lessons. First, it highlights the critical role that policy windows play in policy formation. According to Kingdon, policy-making environments are often characterized by three parallel streams namely the problem, solution, and political stream.^38^ *Problem*s refer to the challenges or issues that are experienced and recognized by stakeholders, including policy-makers and citizen.^38^ In the Kenyan case, the problem stream was characterized by the recognition that maternal health outcomes in the country were consistently poor because of, among others, limited access to priority maternal health services, which in turn was largely a result of financial barriers to access.^2,39^ The *solution* stream refers to policy ideas and interventions that are proposed and debated, sometimes developed, sometimes rejected, and sometimes selected.^38^ In the Kenyan case, multiple solutions had been proposed and debated, key among them being the removal of user fees for maternal health services. The *politics* stream includes demands of interest groups, public opinion, and election processes.^38^ In the Kenyan case, 2013 was a general election year that was characterized by rival political parties making election pledges that included the introduction of health sector policies that would improve the lives of Kenyans. The free maternity policy was one of the key election pledges by the political party that won the general election.

Kingdon proposed that policy windows are created when these three streams, problem, solution and politics, converged and that policy formation occurrs in these instances.^38^ In the Kenyan case, the outcome of the 2013 general election constituted a policy window; the free maternity policy was launched in June 2013, as one of the first policies introduced by the newly elected government, as a fulfilment of their election pledge. This mirrors the experience in other settings where user fee removal policies in general, and those that target maternal and child health services in particular have often been used as election pledges and implemented by governments as a fulfilment of these pledges.^40-42^ It highlights the need for policy entrepreneurs to actively seek, identify and align policy interventions with political interests and processes, and take advantage of policy windows of opportunity in order to advance major health system reforms that would otherwise be difficult to achieve using the more common incremental approaches.

Second, the Kenyan experience highlights the critical role that frontline policy implementers play in policy implementation and the need to adequately engage and communicate to them.^43^ In the Kenyan case, political expediency led to hurried implementation of the free maternity policy, which in turn was characterised by inadequate engagement, and poor communication of the policy to frontline implementers (county health department officials, and health facility managers). Similar experiences have been reported in other settings.^24,44,45^ The engagement of county level stakeholders is especially important in the post 2013 Kenyan context, given the governance system transition from a centralized to a devolved system in which county governments have the mandate to provide health services and own healthcare facilities. In the absence of adequate engagement and communication, sense-making (the process by which actors make meaning and interpret policies, and then act upon it) by frontline policy implementers, labelled street level bureaucrats by Lipsky contributes to policy infidelity. The actions and decisions of street-level bureaucrats, such as Kenyan implementers’ interpretation of the entitlements under the free maternity policy, become policy as experienced by citizens.^46^ In the Kenyan case, frontline policy implementers interpreted the free maternity policy in varied ways that resulted in inconsistency in the range of services covered, and in some cases, charging of user fees.

Third, the implementation of the free maternity policy in Kenya highlights the need for whole system change, as opposed to isolated policy interventions.^47,48^ International evidence shows that while user fee removal policies have the potential to improve the health of citizens, their benefits could be curtailed if they are not accompanied by complementary interventions.^43,49^ Specifically, user fee policies should be accompanied by supply side investments to ensure that healthcare facilities have adequate resources (such as healthcare workers, infrastructure and essential commodities) to match the anticipated increase in demand of healthcare services.^49^ Neglect of supply side capacity strains the health system, often leading to unintended outcomes.^19,21^ For instance, in Burundi, inadequate supply side capacity accompanied by user fee removal led to stock outs of drugs, reduced health worker motivation and poor quality services.^50^ Our findings show despite the increase in access to maternal services, the quality of care of these services was compromised by supply side constraints. In addition, a clear and functional plan to compensate healthcare facilities for revenue loss should be in place.^43^ Irregular and unpredictable free maternity funds disbursements, similar to findings in other countries,^21,51^ exposed health facilities in Kenya to financial constraints that compromised service provision. User fee removal and reimbursement policies should also be accompanied by effective accountability mechanisms to reduce fraud and minimize leakage of resources.

### Recommendations for Policy

Drawing from our findings we make a number of recommendations. First, the Kenyan MoH should develop a formal policy on the free maternity services and an implementation plan that clearly articulates the implementation arrangements of the policy. Secondly, the MoH should engage key stakeholders in the implementation of the free maternity programme, namely the county health departments, and healthcare providers and effectively communicate the policy to ensure that there is clarity on policy content and implementation arrangements. Third, the MoH should ensure that funds disbursement to healthcare providers are timely, and predictable. Related to this, the county governments should review their public finance by-laws, and introduce local legal provisions that will give healthcare providers financial autonomy over funds that are disbursed to them. Fourth, the MoH and county governments should conduct an assessment of the supply side capacity gaps of healthcare providers and make investments to build this capacity to match increased demand under the free maternity services policy. Fifth, the MoH and the county health department should strengthen the accountability mechanisms for the free maternity services policy. Specifically, they should incorporate a mechanism for verification of utilization reports submitted by facilities to minimize fraud and subsequent resource wastage. Further, to increase policy fidelity, monitoring and evaluation mechanisms should be enforced. For instance, the policy fidelity could be improved by supportive supervision and/or audit and feedback mechanisms.

## Acknowledgements

This manuscript is published with the permission of the Director of KEMRI. Funds from the Wellcome Trust (#101082) awarded to JC supported ET. EB is funded by a Wellcome Trust Research Training Fellowship (#107527). Additional funds from a Wellcome Trust core grant awarded to the KEMRI-Wellcome Trust Research Program (#092654) supported this work. Edwine W. Barasa, Sassy Molyneux, and Benjamin Tsofa are members of the Consortium for Resilient and Responsive Health Systems (RESYST). However, the views expressed and information contained in it are not necessarily those of or endorsed by DFID, which can accept no responsibility for such views or information or for any reliance placed on them. The funders and the World Bank had no role in study design, data analysis, decision to publish, drafting or submission of the manuscript. The views expressed in the papers are for the authors and not for the organizations they represent.

## Ethical issues

The KEMRI Scientific and Ethics Review Unit approved this study under KEMRI SSC No. 3087.

## Competing interests

Authors declare that they have no competing interests.

## Authors’ contributions

JC and SM conceptualised the study. ET and EW collected data and performed preliminary analysis. ET and EB contributed to further analysis. ET and EB developed the first draft. All authors contributed to subsequent and final drafts.

## Authors’ affiliations

^1^Health Economics Research Unit, KEMRI Wellcome Trust Research Programme, Nairobi, Kenya. ^2^Institute of Healthcare Management, Strathmore University, Nairobi, Kenya. ^3^KEMRI Wellcome Trust Research Programme, Kilifi, Kenya. ^4^The World Bank, Kenya Country Office, Nairobi, Kenya. ^5^Nuffield Department of Medicine, University of Oxford, Oxford, UK.

## Key messages

**Implications for policy makers**

- User fee removal policies should be matched by supply side capacity strengthening to accommodate the anticipated increase in service utilization without compromising quality and straining health system resources.
- The predictability of funding disbursement should be improved by ensuring that payments to facilities are consistent and timely.
- The introduction of policies should be accompanied by adequate communication and clarity on policy content and implementation arrangement, and effective accountability mechanisms so as to minimize deviations between policy on paper and in practice.
- The engagement of relevant stakeholders, and especially frontline implementers is critical in ensuring that policies are appropriately implemented.

**Implications for the public**

This paper provides recommendations for improving the implementation of the free maternity policy, which if adopted will ensure that the public can uniformly and consistently access the complete set of service entitlements (antenatal care [ANC], deliveries, postnatal care [PNC]) under the programme. Implementing the recommendations will also improve the capacity of health facilities to serve the public and hence promote improved quality of services that the public receives from healthcare facilities.
